# Evaluation of *Ajuga bracteosa* for antioxidant, anti-inflammatory, analgesic, antidepressant and anticoagulant activities

**DOI:** 10.1186/s12906-016-1363-y

**Published:** 2016-09-27

**Authors:** Waqas Khan Kayani, Erum Dilshad, Tanveer Ahmed, Hammad Ismail, Bushra Mirza

**Affiliations:** 1 Department of Botany, The University of Poonch, Rawalakot, Azad Jammu and Kashmir 12350 Pakistan; 2 Department of Bioinformatics and Biosciences, Capital University of Science and Technology, Islamabad, Pakistan; 3 Department of Biochemistry, Faculty of Biological Sciences, Quaid-i-Azam University, Islamabad, 45320 Pakistan; 4 Department of Biochemistry and Molecular Biology, University of Gujrat, 50700 Gujrat, Pakistan

**Keywords:** *Ajuga bracteosa*, Antioxidants, Anti-inflammatory, Phytoecdysteroids, RP-HPLC, Antidepressant

## Abstract

**Background:**

*Ajuga bracteosa* has been extensively used traditionally for the treatment of a variety of diseases. The aim of the study was to scientifically validate the wide-scale exploitation of *A. bracteosa* in folk medicine various in vitro and in vivo assays. Moreover, these activities were related to the intrinsic biologically active phytoecdysteroids of *A. bracteosa*.

**Methods:**

Aerial and root parts of *A. bracteosa* were first extracted separately with chloroform (*Ab*CA and *Ab*CR) and the residue was again extracted with methanol (*Ab*MA and *Ab*MR). Total flavonoid and phenolic contents were assayed as quercetin (QE) and gallic acid equivalents (GAE), respectively. These extracts were analyzed for in vitro antioxidant assessment including DPPH and H_2_O_2_ (% inhibition of free radicals), and reducing power and phosphomolybdenum methods (ascorbic acid equivalents AAE mg/g DW). Further, these extracts were assayed in vivo in separate groups of Sprague–Dawley rats for carrageenan induced rat paw edema inhibition, hotplate antinociception, forced swim antidepression and anticoagulation. Dose of each crude extract and standard drug given to rats was 200 mg/Kg- and 10 mg/10 mL/Kg body weight respectively. Plant extracts and standard drugs were administered orally, 60 min prior to the conduction of assays. Moreover, biologically active phytoecdysteroids were screened in *A. bracteosa* with the help of RP-HPLC.

**Results:**

*Ab*MA represented highest values of flavonoids (QE 1.98 % DW) and phenolic contents (GAE 5.94 % DW), significantly scavenged DPPH radicles (IC_50_ 36.9) and reduced ferric ions with 718.4 mg ascorbic acid equivalent/g (AAE). Highest total antioxidant capacity was expressed by *Ab*MR (927 mg AAE) with an IC_50_ value 19.1 μg/mL. The extracts which were found potent anti-oxidants, were also good at in vivo activities. *Ab*MA significantly reduced edema in all the three hours of treatment (67.9, 70.3 and 74.3 %). *Ab*MA also showed maximum nociceptor suppression in analgesic assay by delaying the time to start licking of paws in rats (57.7 ± 4.9 s). In addition, maximum anti-coagulation was also exhibited by *Ab*MA (89.3 s), while all extracts were found strong antidepressants (≤15.66 s immobility time). Screening of biologically active phytoecdysteroids revealed the presence of 20-hydroxyecdysone (20-HE), makisterone (MKA), cyasterone (CYP) and ajujalactone (AJL). Total phytoecdysteroid content found in *A. bracteosa* was 1232.5 μg/g DW and 20-HE was most abundant (1232.5 μg/g DW) as compared to other phytoecdysteroids.

**Conclusion:**

Based on the tested in vitro and in vivo activities, *Ab*MA was found to be a promising bioactive extract. These activities can be attributed to the intrinsic polyphenols and phytoecdysteroids contents of *A. bracteosa*.

## Background

Stress stimuli (biotic and abiotic) and intrinsic oxygen metabolism generate reactive oxygen species (ROS) as by-products. ROS production inactivates enzymes and damages vital cellular organelles and membranes, consequently causing cancers, aging, chronic inflammation, and plays a role in HIV infection, diabetes etc. [1–3]. ROS also modulate the principal neurotransmitters involved in the neurobiology of depression. Oxidative stress is implicated in depression and anxiety [4–6] and foremost depression is linked to lower levels of several endogenic antioxidant arrays [7]. This indicates that oxidative stress processes might also play a relevant role in depression. ROS are also known to provoke inflammation and associated pain caused by tissue injury [8].

Antioxidants protect biological systems from deleterious effects of ROS by free radicals scavenging mechanism. Antioxidants can mitigate ROS effect by reacting with free radicals, blocking the enzyme generating free radicles and provoking the expression of antioxidant enzymes [9]. Antioxidants include carotenoids, anthocyanidins, catalase, glutathione peroxidase ferritin, superoxide dismutase, ceruloplasmin, catechins, vitamin C, tocopherols vitamin E, glutathione and flavonoids etc. [10, 11]. Synthetic antioxidants like butylated hydroxytoluene (BHT) and butylated hydroxyanisole (BHA) show toxicity to living systems [12]. Moreover, myocardial infarctions caused by intravascular blood clots are major cause of deaths worldwide. Although heparin has been used to treat acute thrombotic disorders as a primary drug, yet it poses some complications in its clinical usage [13, 14]. To cope with these problems, there is an increasing demand of alternative antioxidants and antithrombotic agent (anticoagulants). Medicinal plants have historically been used as primary source of antioxidants [15] and anticoagulants [16].

*Ajuga bracteosa* Wall. ex. Benth. (Family Labiateae) is a perennial plant and is distributed widely in Kashmir and sub-Himalayan tract. It is recommended in Ayurveda to treat numerous ailments [17–19]. Medicinal properties of *A. bracteosa*, such as astringent, anthelmintic, anti-inflammatory and anti-microbial led its use into folk medicine [17]. In a recently reported quantitative ethnobotanic survey of Khyber Pakhtunkhwa (KPK) province of Pakistan, 92 medicinal plants species were listed. Among them, *A. bracteosa* represented highest usage frequency (6) especially as blood purifier, carminative, anti-cough, anti-asthma, anti-jaundice and as a cooling agent [20]. Decoction of its bark is used to cure jaundice and sore throat [21]. Leaves extract of *A. bracteosa* is used as a remedy for acne, pimples, stomach disorders, ear and throats infections and headache. Moreover the extract of leaves of *A. bracteosa* is regarded as blood purifier and cooling agent e.g. effective in diuretic functions [22]. Moreover, it is found to be pharmacologically active against cancer, hypoglycemia, protozoal diseases, spasmodic activity, gastric ulcer and liver fibrosis [23, 24].

Various extracts of *A. bracteosa* has been evaluated by some groups worldwide to assess its pharmacological applications. Ethanolic extract of *A. bracteosa* (70 %: EEAB), when employed over mouse ear edema, resulted in a significant and dose-dependent anti-inflammatory activity at the dose of 0.5 and 1.0 mg/ear [18]. EEAB also resulted in a significant and dose dependent inhibitory effect against acute and chronic arthritic models of albino rats comparable to aspirin [17]. In mice models, CCl_4_ induced hepatic fibrosis was treated with chloroform extract of *A. bracteosa* which shielded the liver from injury by reducing the activity of plasma aminotransferase and inhibiting the hepatic mRNA expression of CD14 and TNF-α [24]. Various extracts of *A. bracteosa* was assessed in Swiss albino mice models for their analgesic potentials in acetic acid-induced writhing test and tail immersion test. Chloroform and aqueous extracts showed significant and dose-dependent analgesic effects at a 200 and 400 mg/Kg, i.p doses [23]. Some extracts of *A. chamaecistu* were analyzed for formalin induced antinociceptive activity in mice. Hexane fraction at 200 mg/Kg body weight of mice (BWM) caused significant analgesic effects on the chronic phase (15–60 min after formalin injection). However, to obtain the same analgesic effects by aqueous and diethyl ether extracts, a dose of 400 mg/Kg BWM was required [25]. Besides these extracts, methanolic extract of *A. bracteosa* induced a strong in vivo anti-nociceptive effect in treated animals at a dose of 500 and 750 mg/Kg BWM in three different analgesic assays [26].

These activities can be attributed to the intrinsic natural products of *A. bracteosa* like phytoecdysteroids, withanolides, clerodanes etc. Phytoecdysteroids are attributed to impart the antioxidant character in *A. bracteosa*. They also regarded to prevent body from infections and depression [27]. Ecdysteroids, major constituents of *A. bracteosa* has been exploited commercially and an ecdysteroid containing adaptogenic preparation (Leventon) was found to increase in anticoagulation potential in athletes [28]. Therefore, the assessment of a variety of extracts of *A. bracteosa* for in vitro antioxidant activities and in vivo anti-inflammatory, analgesic, antidepressant and anticoagulant activities on Sprague–Dawley rats were performed.

## Methods

### Equipment, chemicals and reagents

The solvents were purchased from Sigma Aldrich, GmbH Buchs Switzerland. Whatman filter paper 1 was used to filter the extracts which were concentrated in BUCHI Rotavapor R-200 rotary evaporator. Dried crude extracts were dissolved with the help of Elmasonic Sonicator (E_30-H_ Germany). Salts, standards, chemicals and reagents were purchased from Sigma Aldrich USA, Merck Germany, Panreac Quimica SA Barcelona Spain and BDH Reagents & Chemicals England. Change in absorbance was recorded by UV–VIS Spectrophotometer (Agilent technologies). For hot plate analgesia, Eddy’s hot plate (IKA, Germany) was used. Paw volume was measured by Plethysmometer (Ugo Basile 7140).

### Collection and identification of plant

**Fig. 1:**
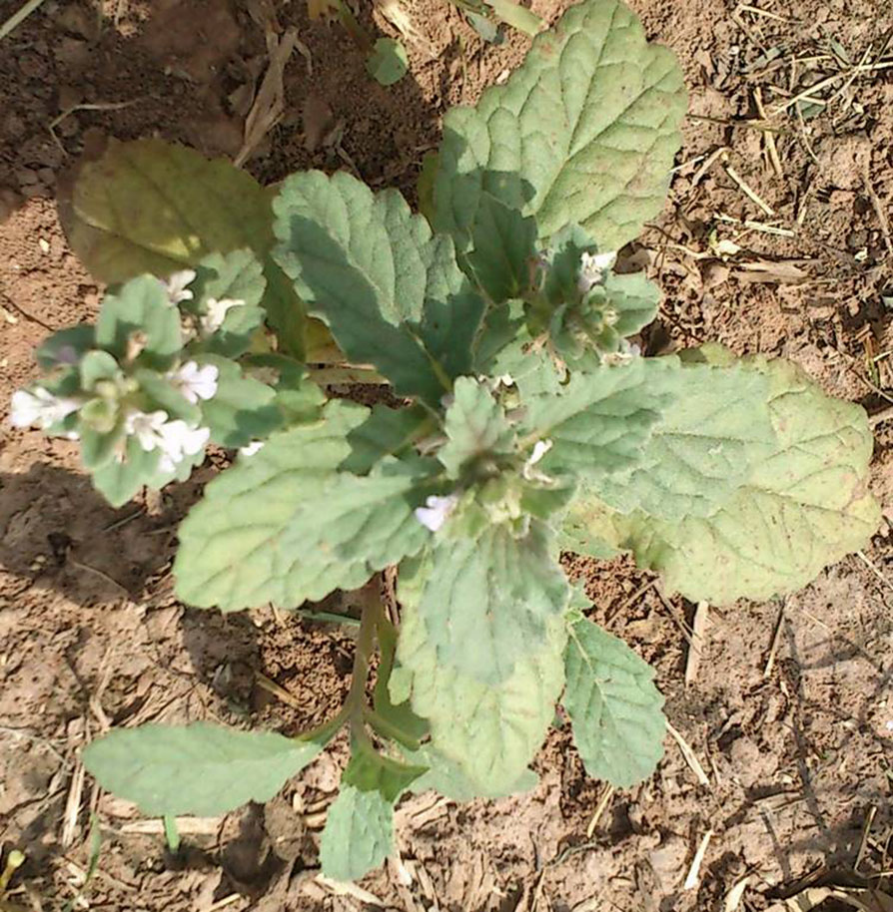
Wild type *Ajuga bracteosa*

*Ajuga bracteosa* Wall. ex Benth. was collected from University Campus of Quaid-i-Azam University (QAU) (Fig. 1). The plant was identified by taxonomist in Plant Sciences department wide a voucher specimen (HMP-460) which was deposited in “Herbarium of medicinal Plants of Pakistan” at QAU Islamabad, Pakistan.

### Preparation of extracts

**Fig. 2:**
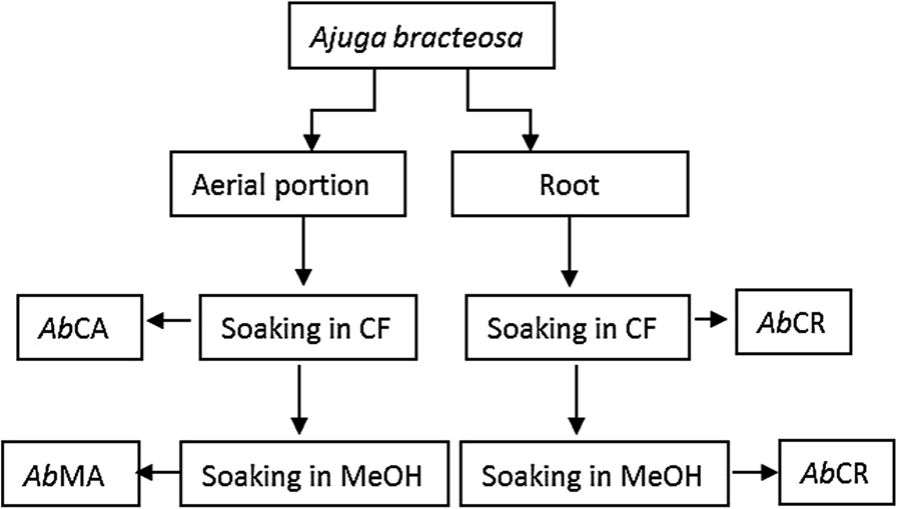
Extraction scheme:*Ab*MR: methanolic extract of root, *Ab*MA: methanolic extract of aerial portion, *Ab*CR: chloroform extract of root, *Ab*CA: chloroform extract of aerial portion

Fresh plant material was rinsed with distilled water and aerial part was separated from root. Both parts were air dried under shade. Extract was prepared by soaking ground plant powder (both parts separately) in chloroform. After 24 h, chloroform extract was filtered with white cotton cloth. The residue was extracted two more times in the same way. Mixture was further filtered and concentrated in rotary evaporator at 40 °C. The subsequent residue was extracted with methanol in the same way as described before. Semi-liquid extracts were dried in fume hood and stored at −20 °C for further usage. The overall scheme of extraction process is shown in Fig. 2.

The names of extracts were abbreviated as; the first two italicized letters represent plant (*Ab, Ajuga bracteosa*), the third letter represents the solvent (M, Methanol; C, Chloroform) and the last letter represent the plant part (A, Aerial portion; R, Root). For example *Ab*MA represents the methanolic extract of aerial parts of *A. bracteosa*.

### Determination of total flavonoid content

Aluminum chloride colorimetric method [29] was used to assay total flavonoid content with some modifications. Briefly, an aliquot of 0.5 mL of various extracts (1 mg/mL) were added to 1.5 mL of methanol, followed by the addition of aluminum chloride (0.1 mL, 10 %), potassium acetate (1 M, 0.1 mL) and distilled water (2.8 mL). This reaction blend was incubated for 30 min at room temperature and its absorbance was recorded with spectrophotometer at 415 nm. The calibration curve (0 μg/mL to 8 μg/mL) was plotted by using quercetin as a standard. The total flavonoids were expressed as mg quercetin equivalent (QE/g) dry weight.

### Determination of total phenolic content

Folin-Ciocalteu reagent method was used to measure total phenolic content [29]. Briefly, an aliquot of various extracts (0.1 mL, 4 mg/mL) was mixed with Folin-Ciocalteu reagent (0.75 mL, 10-fold diluted with dH_2_O). The resultant mixture was left at room temperature and exactly after 5 min sodium carbonate (0.75 mL of 6 %) was added to it. It was followed by a 90 min of reaction time and finally its absorbance was recorded at 725 nm. The standard calibration (0 μg/mL to 25 μg/mL) curve was plotted by using Gallic acid as standard and total phenolic content was expressed as mg Gallic acid equivalent (GAE/g) per gram dry weight of extract.

### In vitro antioxidant assays

Extracts of *A. bracteosa* were analyzed for their antioxidant potential at 1000, 500, 250, 125, 62.5, 31.25, 15.63 and 7.81 μg/mL concentrations (prepared in DMSO). In all the assessment methods, ascorbic acid was used as positive control. For a more reliable conclusion, extracts were assayed by four different antioxidant determination methods.

#### 2, 2- Diphenyl-1-picryl-hydrazyl radical (DPPH) assay

Already reported method was followed for DPPH assay with some modifications [30]. Briefly, 950 μL of methanolic solution of DPPH (3.2 mg/100 mL) was mixed with 50 μL of each extract in eppendorf tubes, mixed and kept in dark at 37 °C for 1 h. Change in absorbance was recorded at 517 nm with spectrophotometer. IC_50_ value was calculated by table curve method and percentage inhibition was measured by following equation:

$$ Percentage\  inhibition = \frac{Absorption\  of\  control- absorption\  of\  samples}{absorption\  of\  control} \times 100 $$

#### Hydrogen peroxide (H_2_O_2_) scavenging assay

Hydrogen peroxide radicle scavenging activity of the extracts was measured according to an established protocol with some modifications [31]. Briefly, a solution of H_2_O_2_ (2 mM) was prepared in phosphate buffer (50 mM, pH 7.4) and 0.3 mL of fresh buffer was mixed with 0.1 mL of various plant extracts separately. It was followed by the addition of H_2_O_2_ solution (0.6 mL) and brief vortexing. The reaction mixture was left for 10 min and absorbance was recorded at 230 nm. The H_2_O_2_ radicle scavenging ability was calculated by the following formula:

$$ \mathrm{Hydrogen}\ \mathrm{peroxide}\ \mathrm{svavenging}\ \mathrm{activity} = \left(1 - \frac{Absorption\  of\  sample}{absorption\  of\  control}\right) \times 100 $$

#### Reducing power

The reducing potential of the extracts was assayed as described before [32]. Plant extracts (0.2 mL of each concentration separately) was added to phosphate buffer (0.5 mL, 0.2 M, pH 6.6) and potassium ferricyanide [K3Fe (CN)6] (1 %), mixed and incubated at 50 °C for 20 min. Further trichloroacetic acid (0.5 mL, 10 %) was added to it and the mixture was centrifuged for 10 min at 3000 rpm. The supernatant (0.5 mL) was mixed with distilled water (0.5 mL) and ferric chloride [0.1 mL of 0.1 % (w/v) FeCl_3_]. After 10 min, change in absorbance was recorded at 700 nm.

#### Total antioxidant activity

Total antioxidant activity was assessed by phosphomolybdenum assay [33]. Briefly, an aliquot of crude extract (0.1 mL) was combined with reagent solution [1 mL, 0.6 M sulphuric acid (H_2_SO_4_), 28 mM sodium phosphate (Na_3_PO_4_) and 4 mM ammonium molybdate (NH_4_)_6_Mo_7_O_24_.4H_2_O]. It was incubated at 95 °C for 90 min and cooled to room temperature. Change in absorbance was measured at 765 nm and antioxidant effect was determined by following equation.

$$ Antioxidant\  effect\ \left(\%\right) = \frac{Absorption\  of\  control- absorption\  of\  samples}{absorption\  of\  control} \times 100 $$

### In vivo assays

#### Test animals, their grouping and dosage

For all in vivo assays, Sprague–Dawley rats (6 weeks; 185–205 g) provided by the NIH Islamabad of either sex were selected. The study protocol for laboratory animal use and care was legitimated by the Institutional Animal Ethics Committee (Quaid-i-Azam University Islamabad). They were maintained and bred in the Animal House at environmental conditions: 12 h light and dark cycles, 25 ± 1 °C temperature and 50 % relative humidity. The rats were kept in stainless steel standard cages under hygienic conditions and fed with an autoclaved feed and water *ad libitum*. Rats were acclimatized for 2 weeks in this environment before the conduction of experiments. Distilled water was used for the oral administration of standard drug and plant extracts in all in vivo assays. Before the conduction of each assay, rats were divided into six groups, weighed and marked with numbers and each group received different treatment administered orally (through feeding tube) 60 min prior to the conduction of assays. First group received only distilled water (blank or negative control) while second group received standard drugs (positive control) at 10 mg/10 mL/ Kg rat body weight concentration. Group 3, 4, 5 and 6 received *Ab*MR, *Ab*MA, *Ab*CR and *Ab*CA respectively at 200 mg/Kg body weight of rats.

#### Anti-inflammatory assay

Crude extracts of *A. bracteosa* were evaluated for their anti-inflammatory activity by carrageenan induced edema assay [34, 35]. For edema induction, 100 μL of 1 % λ-carrageenan, prepared in 0.9 % NaCl was injected sub-plantar into the left hind paw of each rat. The volume of the rat paw was determined before and after injection followed by one hour regular reading from 0 to 4th hour with the help of UGO Basile Plethysmometer (7140) calibrated with electrolyte solution (0.05 % NaCl and 0.3 % surfactant). Data was recorded in triplicate for every interval. Dichlofenac potassium and saline were used as positive and negative controls respectively. The increase in paw volume was determined by minusing the 0 h reading from respective interval reading while % edema inhibition was calculated by following equation.

$$ Perentage\  inhibition=\frac{NC-CE}{NC} \times 100 $$

Where; *NC* = Mean value of edema in negative control

*CE* = Mean value of edema in respective extract

#### Analgesic assay (Hot-plate method)

For the conduction of hot plate analgesia, a modified form of already optimized method [36] was followed. Standard drug, dichlofenac potassium was used as positive control. The animals were placed on Eddy’s hot plate kept at a temperature of 55 °C. Reaction time was documented when licking or jumping response against heat stimuli appeared in rats [37]. Moreover a number of lickings in proceeding 30 s after first lick were also recorded.

#### Antidepressant assay

Forced swim method [38] was used to screen the extracts for their potential as antidepressants. Fluoxetine HCl was used as positive control. The rats were acclimatized in a water filled glass tank (46 cm height, 21 cm diameter and 30 cm depth) maintained at 37 °C. The level of water was adjusted in such a way that rats could support themselves by retaining their tails at bottom. Rats were allowed to move freely in water tank for six minutes to complete their swimming practice. During the conduction of assay, rats were placed in water tank one by one and then after 2 min immobility time calculated with the help of stop watch. Immobility time refers to that time when rats halt all movement except those necessary for survival or to balance their body [39]. It can be calculated by the following amended formula [40].

$$ Immobility\  time=360- mobility\  time $$

#### Anticoagulant assay

Capillary tube experiment was performed to determine the blood clotting activity of the crude extracts [41]. One hour after the oral dosage, the blood was collected in capillary tube by pricking the tails of rats with sterile needles. The blood filled tubes were sealed and placed in water bath at 37 °C. Then after every 10 s small part of tube was broken until the thread formation of fibrin protein started. The clotting time was determined by taking the blood appearance on rat tail as starting point and thread formation as end point.

### Extraction of phytoecdysteroids and RP-HPLC analysis

**Fig. 3:**
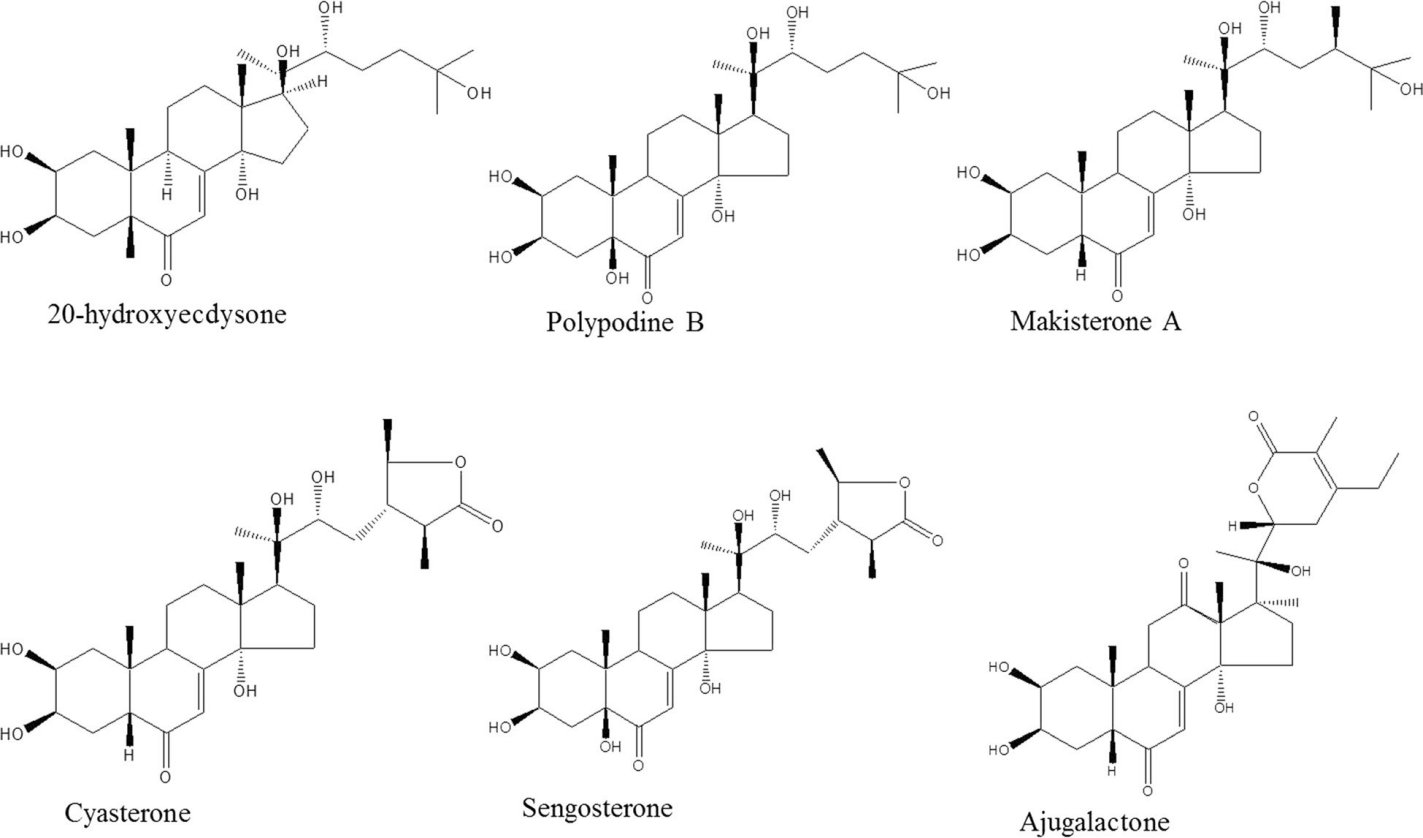
Chemical structures of the used phytoecdysteroids standards

Six ecdysteroids screened in the study were; 20-ydroxyecdysone (20-HE), Ajugalactone (AJL), Sengosterone (SG), Cyasterone (CYP), Polypodine (PoB) and Makisterone A (MKA) (Fig. 3). For the extraction and analytical HPLC of ecdysteroids, already optimized protocol was followed [42].

### Statistical analysis

Crude extracts were assessed in vitro at eight different concentrations were triplicated. The same extracts were assessed in vivo at one concentration and triplicated with three rats in each replicate except analgesic assay which was hexaplicated. Statistical analysis consisted of descriptive statistics using SPSS Statistical Package (version 16.0) and represented as means ± standard deviation.

## Results and discussion

### Total flavonoid and phenolic content

**Fig. 4:**
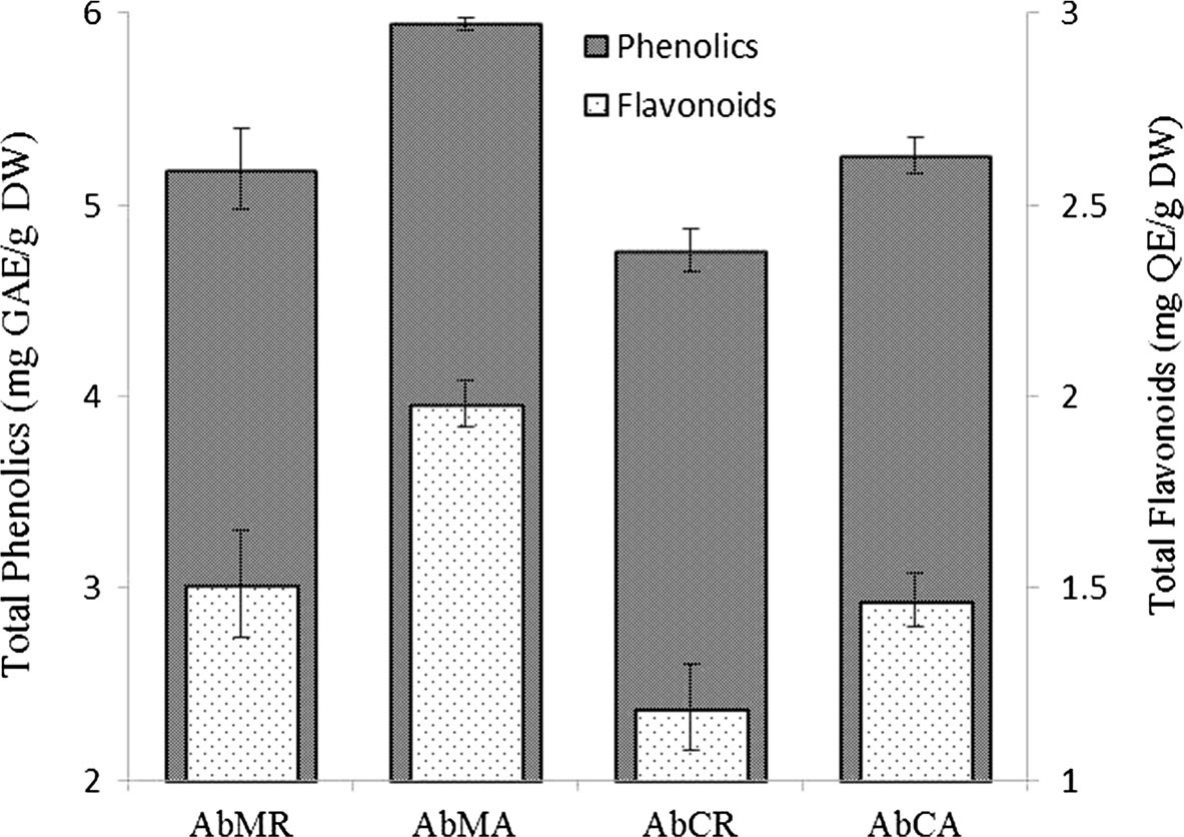
Flavonoids and phenolic contents in different crude extracts of *Ajuga bracteosa*. Total phenolic content was expressed as Gallic acid equivalent (GAE) while total flavonoids were expressed as quercetin equivalent (QE)

Considerable differences were observed among the flavonoids and phenolic contents of various extracts (Fig. 4). *Ab*MA represented highest values of flavonoids (mg QE/g DW 1.98 ± 0.06) followed by *Ab*MR (mg QE/g DW 1.51 ± 0.14) with a significant difference. Results clearly demonstrate that high flavonoid contents are found in methanolic extracts and aerial portions of *A. bracteosa*. Like flavonoids, the highest phenolics content was also found in *Ab*MA (mg GAE/g DW 5.94 ± 1.98), followed by *Ab*CR (mg GAE/g DW 4.76 ± 0.11). In the present study, the levels of flavonoids and phenolic compounds were found to be higher in aerial portion of plant than in roots as reported earlier [43]. Moreover, the solvents of different polarity affect polyphenol content and antioxidant activity [44]. In folk medicines, aqueous extract of aerial parts of *A. bracteosa* is used generally for the treatment of a variety of ailments. We infer polar solvent (methanol) are rich in polyphenols, which might be a reason for the potency of folk use of aqueous extracts.

### Antioxidant assays

Antioxidant behavior of plant extracts can be attributed to their ability to prevent chain initiation, block nonstop hydrogen abstraction, bind to transition metal ion catalysts, peroxides decomposition, reductive competency and radical scavenging [45, 46]. Antioxidant activity measured by one method cannot show the true antioxidant potential of any substance, because one method of quantification relay on only one mechanism [47]. Commonly used antioxidant assays are; DPPH assay, H_2_O_2_ quenching assay, reducing power, total antioxidant activity etc. [48]. In the present study we conducted four antioxidant assays, in order to evaluate a broad range of antioxidant activity of *A. bracteosa* extracts.

#### DPPH assay

**Fig. 5:**
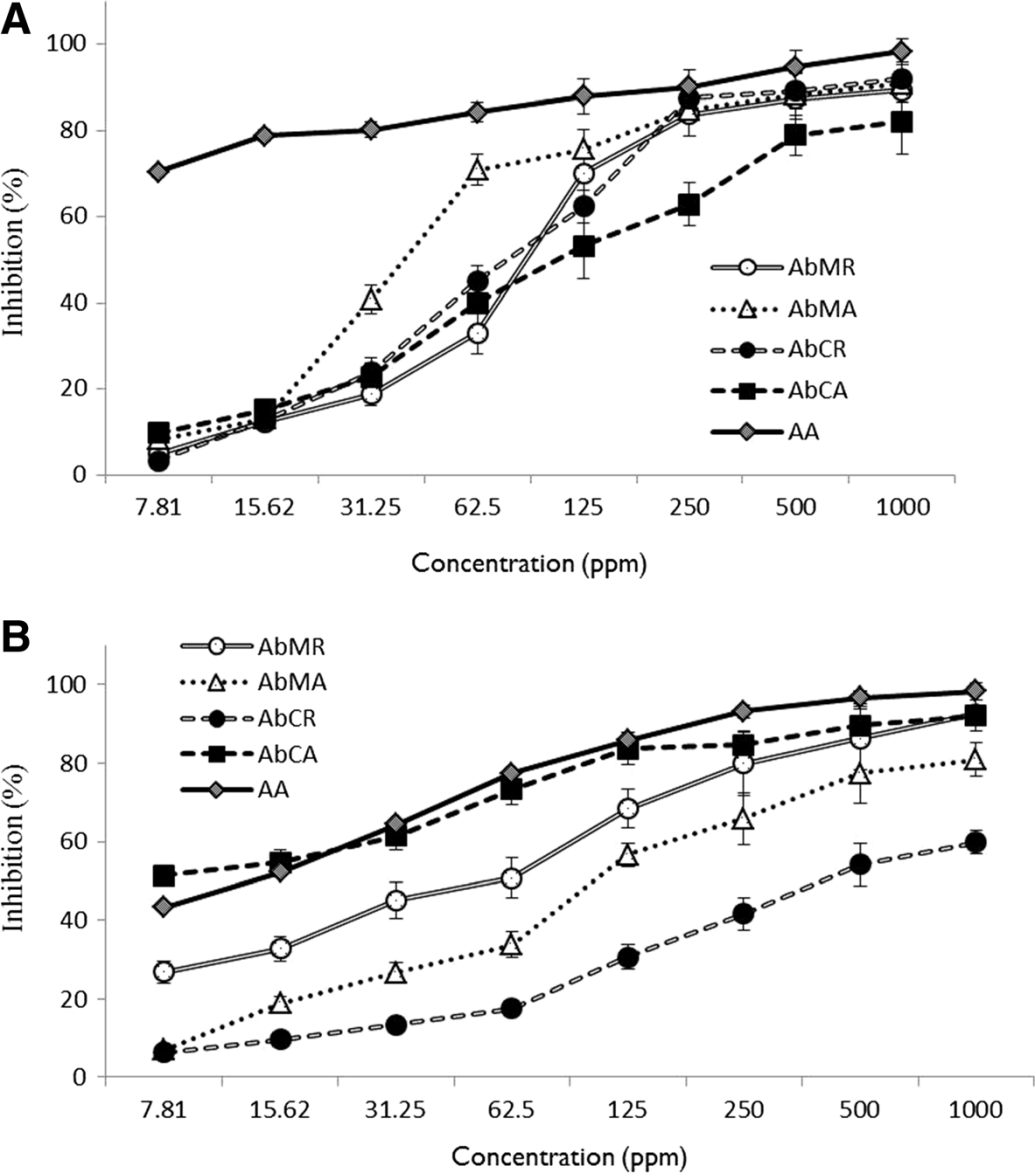
Percentage scavenging of DPPH (**a**) and H_2_O_2_ (**b**) by crude extracts of *A. bracteosa* against ascorbic acid (AA). Data is expressed as mean ± SD

Extracts which can donate hydrogen atom to DPPH radical are considered good antioxidants. DPPH free radical scavenging was found highest for *Ab*MA (Fig. 5a). *Ab*MA, at 31.25 μg/ mL, 62.5 μg/ mL and 125 μg/ mL represented 40.7 ± 3.3, 70.74 ± 3.6 % and 75.52 ± 4.8 % free radical scavenging activity respectively, which is the higher among all the tested extracts. *Ab*MA represented promising DPPH free radical scavenging activity and highest flavonoids and phenolic contents. As flavonoids and phenols are cogent free radical scavengers [49], there seems a strong positive correlation between DPPH free radical scavenging activity and flavonoids and phenolic compounds of methanolic extracts of aerial parts.

#### Hydrogen peroxide scavenging (H_2_O_2_) activity

There is a decrease in absorbance of H_2_O_2_ upon its oxidation. *Ab*CA represented significantly high H_2_O_2_ free radical scavenging activity at all the tested concentrations (Fig. 5b). It represented 51.29 ± 2.2 % and 92.08 ± 4 % scavenging of H_2_O_2_ radicals at 7.81 and 1000 μg/mL concentration respectively. H_2_O_2_ is a free radical that rapidly decomposes into oxygen and water. It can also produce hydroxyl radicals that can initiate lipid peroxidation (LPO) and cause DNA damage in the body. To neutralize it, phenolic compounds donate electron to H_2_O_2_ to generate water [50]. It can be suggested that phenolic content found in the extract offered electron to H_2_O_2_.

#### Reducing power assay

**Fig. 6:**
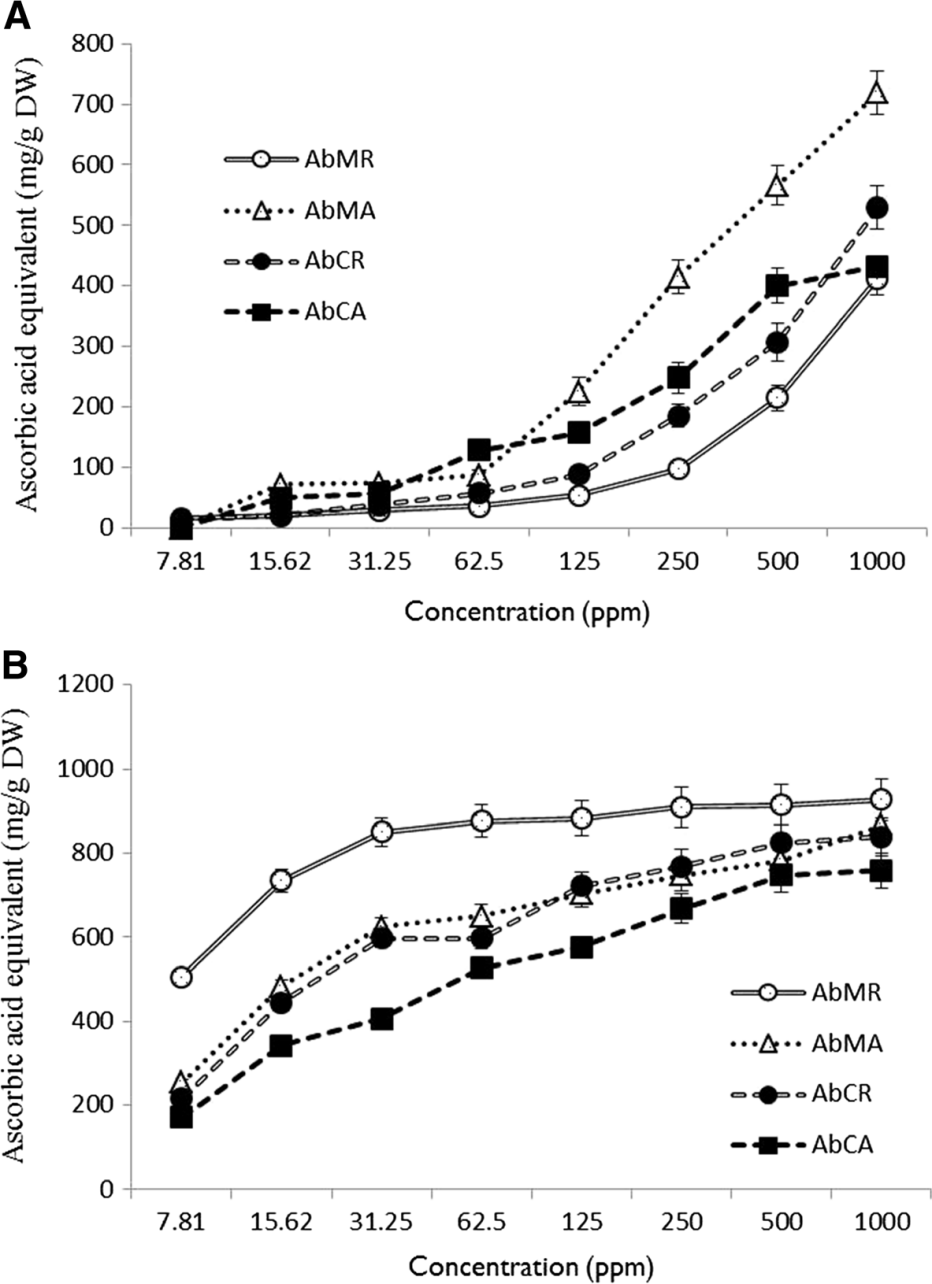
Reducing power (**a**) and total antioxidant activity (**b**) of crude extracts of *A. bracteosa* expressed as ascorbic acid equivalent (AAE). Data is expressed as mean ± SD

Antioxidants alter oxidation state of iron by donating electron and reducing ferric ion to ferrous ion (Fe + 3 to Fe + 2) [51]. Highest reducing power was exhibited by *Ab*MA with 718.4 ± 36 mg ascorbic acid equivalent/g measured at 1000 μg/mL concentration. It was followed by *Ab*CR 529.1 ± 35.9 mg ascorbic acid equivalent/g measured at 1000 μg/mL concentration. *Ab*CA and *Ab*MA represented in the same way with least reducing powers. This assay revealed *Ab*MA as having potent reducing power and antioxidant activity at 15.62 to 1000 μg/mL concentration as compared to the other extracts (Fig. 6a).

#### Phosphomolybdenum method

In phosphomolybdenum assay, Mo (VI) is reduced to Mo (V) by the analyte extracts and subsequent formation of a green phosphate Mo (V) complex [48]. Total antioxidant activity determination gave highest value for *Ab*MR i.e. 506.14 ± 16.68 and 927 ± 50.62 mg ascorbic acid equivalent/g (AAE) at 7.81 and 1000 μg/mL concentration. It was followed by *Ab*MA and *Ab*CR with an AAE of 866 ± 47.94 and 838 ± 46.40 mg/g respectively at 1000 μg/mL concentration. This method represented *Ab*MR as valuable antioxidant extract (Fig. 6b). The logic behind this behavior may be explained by the structure of the antioxidants that can donate electrons/hydrogen to free radicals [52].

#### Comparison of antioxidant assays

When IC_50_ values of the extracts were compared to evaluate antioxidant capabilities, IC_50_ value of *Ab*MA in DPPH radical scavenging (36.9 μg/mL) and reducing power assay (1.5 μg/mL) were found promising. *Ab*MR also represented significant IC_50_ value in phosphomolybdenium radical reduction (19.1 μg/mL). Chloroform extract of aerial parts only exhibited significant activity in hydrogen peroxide assay. The results of antioxidant assays show the scavenging of free radicals or reduction of chemicals in concentration dependent manner. Our results clearly depicted that methanolic extracts of aerial part of *A. bracteosa* possess better antioxidant potential than its chloroform extracts and that of root portion (Table 1). The literature also suggests the polarity of the solvent as an important parameter to extract the antioxidant compounds from a plant [53]. Although the principal compounds of *A. bracteosa* harboring antioxidant activity are not known, polyphenols got attention due to their antioxidant potential especially free radical scavenging [54]. Phenolic compounds are competent source for free radical scavenging [55]. Polyphenolic compounds are extensively present in plant derived food products, and they confer additional antioxidant attributes. Higher phenolic contents of the medicinal plants take priority over other attributes to treat different diseases [56]. It is considered that a positive correlation exists between phenolic contents and free radical scavenging [44]. The plants containing abundant flavonoids are considered to be a probable source of natural antioxidants [54]. Besides polyphenolic compounds, phytoeceysteroids are also regarded as strong antioxidants and majority of them are reported and isolated from *A. bracteosa* (discussed in detail in section 3.3.5).

**Table 1:** Comparison of IC_50_ values in different antioxidant assays

Plant extract	DPPH	H_2_O_2_	Phosphomolybdenum	Reducing power
*Ab*MR	73.4	44.6	19.1	0.9
*Ab*MA	36.9	96.6	65.3	1.5
*Ab*CR	70.6	434.7	59.9	1.1
*Ab*CA	104.4	8.8	106.5	0.9
AA	0.4	12.7	3.3	2.1

Reducing power assay describes absorbance intensity as a measurement of antioxidant activity

*AA* ascorbic acid

Sometimes plants with high phenolic and flavonoid content exhibit low antioxidant activity and *vice versa*. We find *Ab*MA in DPPH and reducing power assay while *Ab*MR in phosphomolybdenum assay as potent extract to scavenge free radicals. These extracts also possess highest amounts of flavonoids and phenolic contents. But when we compare these extracts to *Ab*CA in H_2_O_2_ assay, it represented a drastically low IC_50_ value (1.5 μg/mL) and low flavonoids and phenolic contents. This indicates that the antioxidant potential of pant extract analyte does not always dependent on total amount of polyphenolic compounds only [57].

### In vivo assays

#### Anti-inflammatory assay

**Fig. 7:**
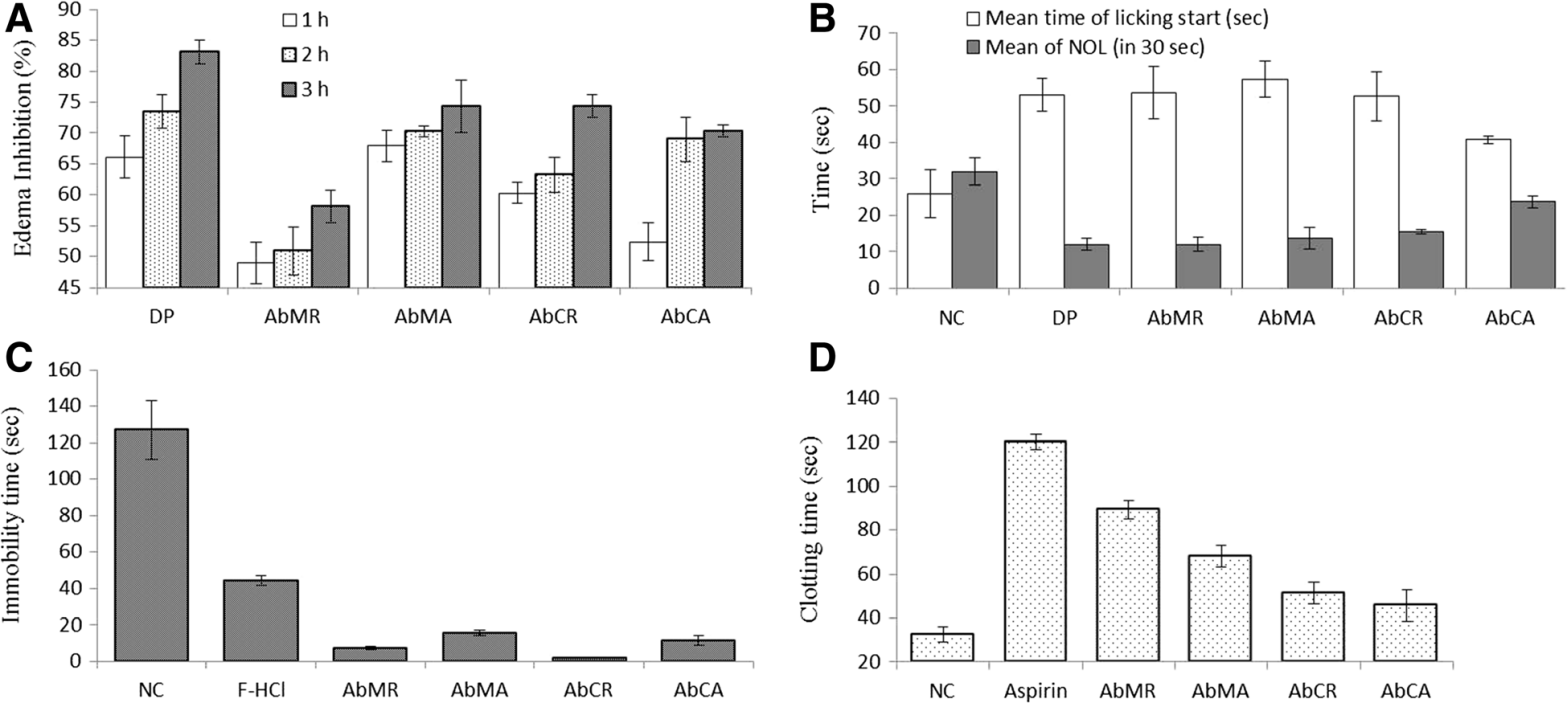
In vivo assays of various crude extracts of *A. bracteosa.* **a** Anti-inflammatory assay, **b** Analgesic assay, **c** Anti-depressant assay and **d** Anti-coagulant assay. NC = negative control; DP = Diclofenac Potassium; NOL = Number of lickings; F-HCl = Fluoxetine HCl. Data is expressed as mean ± SD

Paw edema is an inflammation induced by carrageenan and a decrease in rat paw volume is an indication of anti-inflammatory effects. Test samples represented maximum carrageenan induced rat paw edema inhibition after 3^rd^ hour of treatment (Fig. 7a). Among all the extracts, *Ab*MA performed much better in edema inhibition at all the three hours after treatment at 200 mg/Kg dose (67.9 ± 2.6 %, 70.3 ± 0.9 % and 74.3 ± 4.3 %). Edema inhibition at 3^rd^ hour of treatment is comparable to *Ab*CR (74.4 ± 1.8 %). We suggest that phytoecdysteroids present in *A. bracteosa* are responsible for this activity. These results are supported by a previous study [58] where authors argued that anti-inflammatory activity of *Pfaffia iresinoides* and *Polypodium decumanum* are due to the presence of phytoecdysteroids in these plants. Phytoecdysteroids are reported hepatoprotective being able to regenerate the damaged liver [59]. Phytoecdysteroid improves the cure of wounds or burns [60] and several commercial cosmetics. They inhibit psoriasis activity and are used in cosmetics [61].

#### Analgesic assay

Paw licking in rats is an indication of pain caused by burning from hotplate. Hot plate analgesia assay revealed that *Ab*MA represented maximum analgesic value in delaying the mean time of start of licking (57.7 ± 4.9 s) by suppressing the nociceptors activity in paws (Fig. 7b). *Ab*MR also exhibited a delay in start of licking time (53.7 ± 7.2 s). Good analgesic agents/extracts cause suppression of nociceptors and represent minimum number of lickings. *Ab*MR displayed least number of lickings in 30 s i.e. 12 ± 1.2 s. Depending on the results, both *Ab*MA and *Ab*MR thus were found as beneficial analgesic candidates. Phytoecdysteroids are attributed for the analgesic activity of *A. bracteosa*. Our speculation can be supported by another study [27] where *Pfaffia iresinoides* and *Helleborus* species are regarded to contain active analgesic principle i.e. phytoecdysteroids for which they are capable to relieve pain.

#### Antidepressant assay

Immobility time of a rat in force swim test is an indication of stress and anxiety. The rats were fairly active after oral dose of the extracts. *Ab*CR represented itself as a good antidepressant (2 ± 0.35 s) candidate followed by *Ab*MR (7.3 ± 2.08 s), *Ab*CA (11.66 ± 2.51) and *Ab*MA (15.66 ± 1.52) (Fig. 7c). All potential antidepressant agents increase the mobility time of the rats in forced swim antidepressant assay [62] This strong antidepressant activity of *A. bracteosa* can be linked to the presence of phytoecdysteroids in it. Our findings are supported by previous studies where phytoecdysteroids are reported to be responsible antidepressants in *Leuzea carthamoides* and *Sida carpinifolia* plant species [58]. Lack of endogenous antioxidants results in oxidative stress in depressive disorders [7] and antidepressant potential of phytoecdysteroids can be further explained due to their antioxidant properties.

#### Anticoagulant assay (Clotting time determination)

Extracts of medicinal plants play their haemostatic role being anti-infective, wound healer, and antineoplastic. Fibrin sealants start coagulation cascade when delivered to the bleeding sites and thrombin converts fibrinogen into fibrin to solidify the whole mixture [63]. *Ab*MA represented significantly better inhibitory effects on coagulation activity (Fig. 7d). It delayed coagulation from 32.66 ± 3.51 s (negative control) to 89.3 ± 4.04 s followed by *Ab*MA (68.33 ± 5.03 s). Chloroform extracts remained least effective in anticoagulation property. Herbal resources offer a safe anticoagulant treatment. *Dichrocephala intergrifolia* is used as an effective anticoagulant agent in Cameroon where 95.3 % interviewed patients reported its safety. A Turkish herbal extract (Ankaferd Blood Stopper) is recently approved for the management of external hemorrhage and dental surgery bleeding to reduce coagulation time effectively [64]. Anticoagulation activity of *A. bracteosa* can be attributed to phytoecdysteroid content. This speculation is supported by a previous report in which the researcher [28] administered an adaptogenic preparation containing ecdysteroid (Leveton) to the athletes for 20 days. Besides an increase in working potential of athletes, a significant decline in the blood coagulation was found.

#### Comparison of in vivo assays

Anti-inflammatory agents are usually considered to possess analgesic activities too. In the present study, methanolic extract of aerial portion (*Ab*MA) was found to be a potent extract possessing anti-inflammatory as well as analgesic activities. Moreover, the same extract delayed the coagulation time of rat blood. Besides this, all the extracts exhibited enormous anti-depressant activities. In vivo assays showed their activity in concentration dependent manner. Antidepressants are being used as analgesics for various pain related disorders and their analgesic activity is well recognized [65]. Antidepressants may also benefit the patients having depression and inflammatory pains. Fluoxetine, a standard antidepressant drug is found to significantly decrease inflammation in carrageenan induced rat paw oedema [65]. The anti-inflammatory activity of *A. bracteosa* can be attributed to the presence of phytoecdysteroids and withanolides. Dinan and Lafont reported many studies which describe the wide-scale pharmacological applications of phytoecdysteroids on mammals [27, 58, 66–69]. Evaluation of ethanolic extract (70 %) of *A. bracteosa* is reported expressing a promising anti-inflammatory activity probably mediated through inhibition of cholinesterase enzymes I and II (COX-1 and COX-2). Further 6-deoxyharpagide (isolate of the same extract) exhibited significant COX-2 inhibition [18]. Moreover, withanolides isolated from *A. bracteosa* Bractin A, B and Bractic acid displayed inhibitory potential against enzyme lipoxygenase (LOX), while four of diterpenoids were found to inhibit COX enzymes in a concentration-dependent manner [70]. Based on in vivo studies mentioned here, methanolic extract of aerial portion of *A. bracteosa* hence proved to as an elixir.

In this study we for the first time report the hotplate analgesic assay of *A. bracteosa*. In previous studies, acetic acid-induced writhing test and tail immersion test of chloroform and water extracts (200 and 400 mg/Kg, i.p.) showed significant and dose-dependent analgesic effects probably mediated through opioid receptors [23]. We found all the tested extracts especially *Ab*MR and *Ab*MA exhibiting highly significant analgesic effects at 200 mg/Kg *per os* concentration. It is suggested that the mechanism of this extract may be linked partly to inhibition of LOX and/or COX in peripheral tissues decreasing prostaglandin E2 synthesis and interfering with the mechanism of transduction in primary afferent nociceptor [23]. Antidepressants are also known to possess intrinsic antinociceptive activity. Antidepressants by inhibiting the uptake of monoamines lead to increased amount of noradrenaline and serotonin in the synaptic cleft causing reinforcement of descending pain inhibitory pathways [71].

### Phytoecdysteroids profiling

**Fig. 8:**
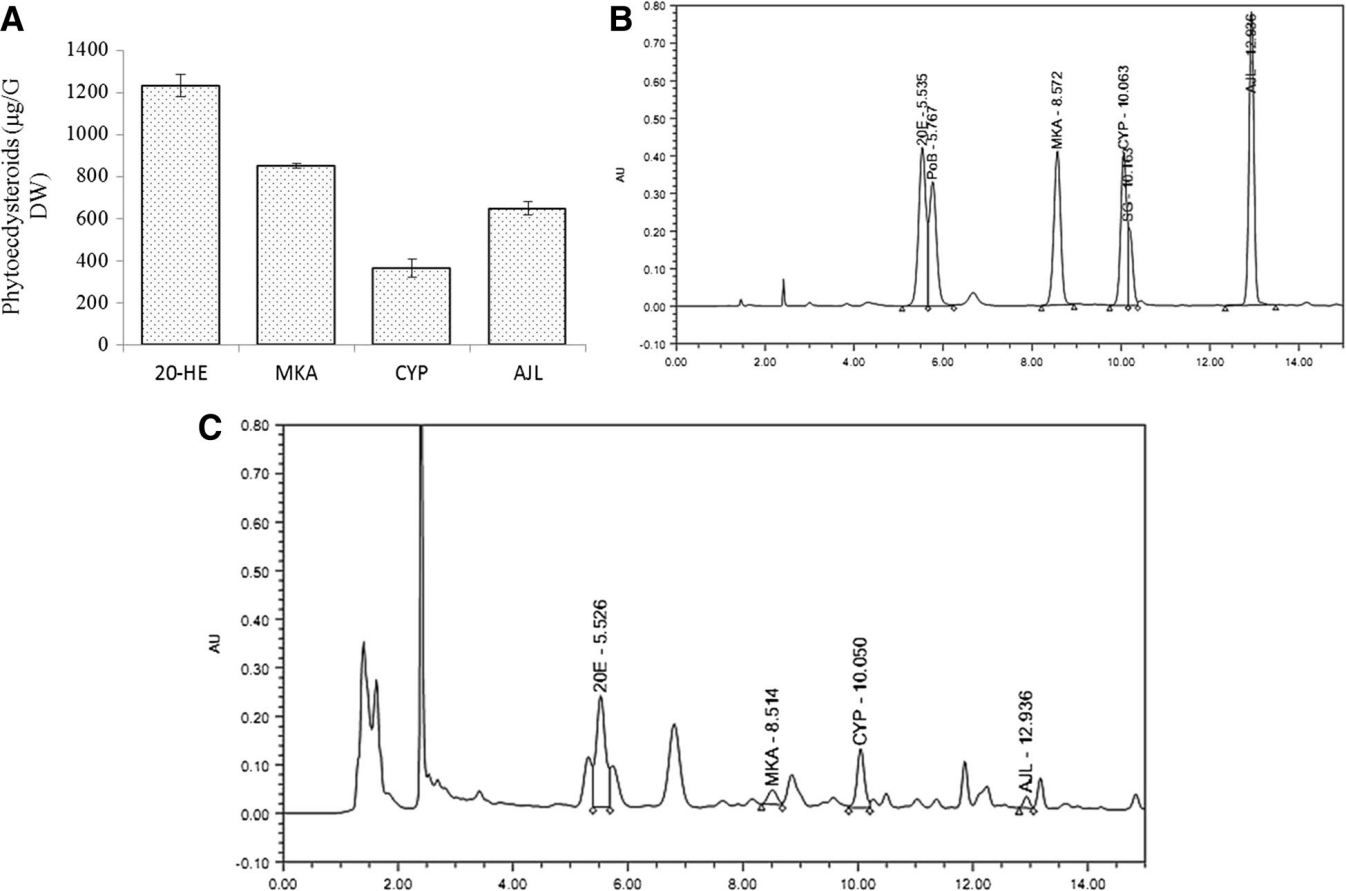
Phytoecdysteroid profiling in *Ajuga bracteosa*. **a** Quantification of phytoecdysteroid content **b** Elution patternof standard phytoecdysteroids and **c** detected phytoecdysteroids. 20-HE; 20-hydroxyecdysone, PoB;Polypodine, MKA; Makisterone; CYP; Cyasterone, SG; Sengosterone, AJL; Ajugalactone

Among the six tested phytoecdysteroids, four were successfully detected in *A. bracteosa*: 20-hydroxyecdysone (20-HE), makisterone (MKA), cyasterone (CYP) and ajujalactone (AJL). Among naturally occurring phytoecdysteroids, 20-HE is most studied and most abundant. (1232.5 μg/g DW) (Fig. 8a-c) and total phytoecdysteroid content detected was 3098 μg/g DW. It is remarkable that certain medicinal plants used in traditional Chinese medicine have high ecdysteroid content. Studies conducted on polar extract of *Serratula coronata* resulted in a significant inhibition of lipid peroxidation (LPO) and phytoecdysteroids and flavonoids present in this extract were suspected for this activity. The fraction rich in ecdysteroids also displayed a significantly higher LPO inhibition in the enzyme-dependent system (10-fold) and in enzyme independent system (two-fold) than α-tocopherol acid succinate [72]. This idea is further supported by the repeated doses of phytoecdysteroids which remarkably regenerated erythrocytes and raised hemoglobin levels in rats [73]. 20-HE was tested on mitochondrial fraction and found oxidizing lipid free radicals in concentration-dependent fashion [74]. It is found that 20-HE successfully terminated the oxidation of lipid free radicals and it showed a double antiradical effect than vitamin D3 (cholecalciferol) and hydroquinone at 8 μM concentration [75, 76]. In another study, 20-HE at a concentration ranging from 10^−6^ to 10^−3^ M also exhibited antiradical and antioxidant properties comparable to that of the known inhibitors of lipid peroxidation: diethyl para-phenylenediamine and ethylenediaminetetraacetate [77].

## Conclusion

We for the first time report a broad range of in vitro and in vivo activities of methanolic and chloroform extract of different parts of *A. bracteosa*. Methanolic extract of aerial parts of plant represented promising in vitro antioxidant and in vivo anti-inflammatory, analgesic, antidepressant and anticoagulant properties and can be suggested as a potent elixir. These activities can be linked to intrinsic active compounds like phytoecdysteroids which are found in highest amounts in methanolic extracts.

## Abbreviations

20-HE: 20-hydroxyecdysone
AAE: Ascorbic acid equivalents
*Ab*CA: *A. bracteosa* chloroform extract
*Ab*CR: *A. bracteosa* chloroform root extract
*Ab*MA: *A. bracteosa* methanolic extract
*Ab*MR: *A. bracteosa* methanolic root extract
AJL: Ajujalactone
BHA: Butylated hydroxyanisole
BHT: Butylated hydroxytoluene
BWM: Body weight of mice
CYP: Cyasterone
DMSO: Dimethyl sulfoxide
DPPH: 2 2- Diphenyl-1-picryl-hydrazyl
DW: Dry eight
EEAB: Ethanolic extract of *A. bracteosa*
GAE: Gallic acid equivalents
KPK: Khyber Pakhtunkhwa
MKA: Makisterone
QE: Quercetin
ROS: Reactive oxygen species
RP-HPLC: Revrs phase high perormane liui homtograpy
